# Influence of Social Media on Applicant Perceptions of Anesthesiology Residency Programs During the COVID-19 Pandemic: Quantitative Survey

**DOI:** 10.2196/39831

**Published:** 2023-06-29

**Authors:** Tyler Dunn, Shyam Patel, Adam J Milam, Joseph Brinkman, Andrew Gorlin, Monica W Harbell

**Affiliations:** 1 Department of Anesthesiology and Perioperative Medicine Mayo Clinic Phoenix, AZ United States; 2 Mayo Clinic Alix School of Medicine Mayo Clinic Phoenix, AZ United States; 3 Department of Orthopedic Surgery Mayo Clinic Phoenix, AZ United States

**Keywords:** anesthesiology residency, application, COVID-19 pandemic, social media, impact, residency, anesthesia, anesthesiology, pandemic, effectiveness, restrictions, barriers, rotations, visits, interviews, applicants, perception, students, program

## Abstract

**Background:**

Social media may be an effective tool in residency recruitment, given its ability to engage a broad audience; however, there are limited data regarding the influence of social media on applicants’ evaluation of anesthesiology residency programs.

**Objective:**

This study evaluates the influence of social media on applicants’ perceptions of anesthesiology residency programs during the COVID-19 pandemic to allow programs to evaluate the importance of a social media presence for residency recruitment. The study also sought to understand if there were differences in the use of social media by applicant demographic characteristics (eg, race, ethnicity, gender, and age). We hypothesized that given the COVID-19 pandemic restrictions on visiting rotations and the interview process, the social media presence of anesthesiology residency programs would have a positive impact on the recruitment process and be an effective form of communication about program characteristics.

**Methods:**

All anesthesiology residency applicants who applied to Mayo Clinic Arizona were emailed a survey in October 2020 along with statements regarding the anonymity and optional nature of the survey. The 20-item Qualtrics survey included questions regarding subinternship rotation completion, social media resource use and impact (eg, “residency-based social media accounts positively impacted my opinion of the program”), and applicant demographic characteristics. Descriptive statistics were examined, and perceptions of social media were stratified by gender, race, and ethnicity; a factor analysis was performed, and the resulting scale was regressed on race, ethnicity, age, and gender.

**Results:**

The survey was emailed to 1091 individuals who applied to the Mayo Clinic Arizona anesthesiology residency program; there were 640 unique responses recorded (response rate=58.6%). Nearly 65% of applicants reported an inability to complete 2 or more planned subinternships due to COVID-19 restrictions (n=361, 55.9%), with 25% of applicants reporting inability to do any visiting student rotations (n=167). Official program websites (91.5%), Doximity (47.6%), Instagram (38.5%), and Twitter (19.4%) were reported as the most used resources by applicants. The majority of applicants (n=385, 67.3%) agreed that social media was an effective means to inform applicants, and 57.5% (n=328) of them indicated that social media positively impacted their perception of the program. An 8-item scale with good reliability was created, representing the importance of social media (Cronbach α=.838). There was a positive and statistically significant relationship such that male applicants (standardized β=.151; *P*=.002) and older applicants (β=.159; *P*<.001) had less trust and reliance in social media for information regarding anesthesiology residency programs. The applicants’ race and ethnicity were not associated with the social media scale (β=–.089; *P*=.08).

**Conclusions:**

Social media was an effective means to inform applicants, and generally positively impacted applicants’ perception of programs. Thus, residency programs should consider investing time and resources toward building a social media presence to improve resident recruitment.

## Introduction

Residency applicants and physicians, in general, are accustomed to using electronic and social media resources for career opportunities [1-4]. A study from 2003 showed that 79% of applicants used a program’s website to decide where to apply and about a third used information from the website to help create a match list [5]. Over the last 2 decades, the use of the internet to gather information about programs is universal among applicants [6,7]. Social media has become an emerging tool in the web-based realm, with about half of the applicants using at least 1 social media platform to research potential programs in 2014 [8]. It has been reported that only about 15% of residency programs retain a social media presence, despite it being a commonly accessed source by applicants [9].

The COVID-19 pandemic had a significant impact on the 2020-2021 residency application cycle for both anesthesiology residency programs and applicants. Traditionally, visiting medical student rotations and in-person activities during the interview process have helped residency programs to evaluate and recruit potential applicants while also allowing applicants to evaluate programs firsthand [10]. However, due to COVID-19 pandemic restrictions, these opportunities were limited. As a result, social media may have played a crucial role for not only residency programs but also applicants going forward in the anesthesiology match process.

There are limited data regarding the impact of social media on residency recruitment. Several studies have shown that there has been a growth in social media use associated with the pandemic including 40 of 76 anesthesiology residency–associated social media accounts having been created after March 2020 [9]. A recent study found that a majority of anesthesiology residency applicants felt social media had at least partially influenced their assessment of programs [6]. Similar findings were found in a study of 650 orthopedic surgery applicants during the COVID-19 pandemic; 60.6% of applicants agreed that social media positively affected their perception of the associated program [11]. This study evaluates the use of social media in the application process for anesthesiology residency programs during the COVID-19 pandemic and also sought to understand if there were differences in the use of social media by applicant demographic characteristics (eg, race, ethnicity, gender, and age), as this has not been reported in the literature. A secondary aim was to develop a novel scale to assess the importance of social media in the application process. We hypothesized that given the COVID-19 pandemic restrictions on visiting rotations and the interview process, the social media presence of anesthesiology residency programs would have a positive impact on the recruitment process and be an effective form of communication about program characteristics. The findings from this study will inform programs of the overall importance of a social media presence for their residency recruitment as well as explore any specific factors such as gender, age, race, or ethnicity that may have differing opinions to allow programs to address recruitment gaps and strengthen their overall applicant pool.

## Methods

### Design

Email addresses of all anesthesiology residency applicants who applied to the authors’ program were obtained from electronic residency application service and were sent a link to the web-based survey in October 2020, included in the Multimedia Appendix 1. This study adheres to the CHERRIES (Checklist for Reporting Results of Internet e-Surveys), which is included in the Multimedia Appendix 2.

### Ethical Considerations

The study was deemed exempt by the Mayo Clinic Institutional Review Board. The survey participants were informed that the survey was administered by Mayo Clinic for the purpose of evaluating the influence of social media on applicants’ perceptions of anesthesiology residency programs during the COVID-19 pandemic. The survey collected no identifying information and included questions regarding subinternship rotation completion, social media resource use, social media impact, and general demographics. Participants were informed that the survey was voluntary and anonymous. The survey had 20 items, and we estimated it would take approximately 5 minutes to complete. No personal information was stored, and the survey data remained on Qualtrics servers.

### Development and Pretesting

The survey was created by the authors and was distributed via Qualtrics. The survey was tested by 3 research team members prior to distribution.

### Recruitment Process and Description of the Sample Having Access to the Questionnaire

This was a closed survey. All contacts with respondents were made via the internet. Email addresses of all anesthesiology residency applicants who applied to the authors’ program were obtained from electronic residency application service.

### Survey Administration

A link to the survey was sent via email, and the responses were automatically captured via Qualtrics between October and December 2020. The questions were not randomized, and there was no adaptive questioning. There were 5 questionnaire items per page, with 20 in total over 4 pages.

### Response Rates and Preventing Multiple Entries From the Same Individual

Cookies were not used to assign a unique user identifier to each client computer. IP addresses were tracked to prevent duplicated responses within the survey period and to calculate correct response rates. Duplicate entries were avoided by preventing users with the same IP address access to the survey. It was an open survey in which recipients of the survey link were able to complete the survey.

### Analysis

Only complete surveys were able to be submitted and subsequently analyzed. A time cutoff of 90 seconds was used for data analysis. This was determined based on the average time needed to complete the survey during development and testing. We are assuming that the data were missing at random, and a complete case analysis was used for analyses.

Descriptive statistics (ie, frequencies and sample sizes) were reported for survey items (SPSS Statistics for Windows, IBM Corp). Survey items were also stratified by race, ethnicity, and gender; chi-square tests were used to examine the differences in perceptions of social media by race, ethnicity, age, and gender. We created a scale representing the importance of social media via the following process: we first collapsed the 5-point Likert scale (eg, strongly agree to strongly disagree) into a 3-point Likert scale (eg, strongly or somewhat agree; neither agree or disagree; and strongly or somewhat disagree) given small cell sizes. We then performed polychoric correlations with the 10 items. There was 1 survey question (social media accounts will have less of an impact on applicant perceptions during feature application cycles not limited by the COVID-19 pandemic) that had a negative correlation with 7 of the other survey items; this question was reverse coded (eg, strongly disagree to strongly agree). Mplus was used to identify factors from the 10 survey items, and 8 items were consistently loaded together in an exploratory factor analysis model and were used to create a scale. Internal consistency reliability of the scale was assessed with Cronbach α. The 8-item factor was regressed on applicant demographic characteristics (age, gender, race, and ethnicity) in Mplus using a structural equation model (SEM), and standardized betas were reported to measure the association between demographic characteristics and the social media factor. To evaluate the SEM fit, root-mean-square error of approximation (RMSEA), comparative fit index (CFI), and Tucker-Lewis index were used. The data were considered a good fit when RMSEA values were ≤0.05, CFI values were ≥0.95, and TLI values were ≥0.90. *P* values were considered significant when ≤.05.

The majority (88%) of respondents answered all questions in the survey; missingness did not vary by age category, race, ethnicity, or gender (*P*>.05).

## Results

### Demographics

The survey was sent out to 1091 individuals, and 640 unique responses were recorded for a response rate of 58.6%. Approximately half of the respondents were non-Hispanic White (n=288, 50.3%,), followed by Asian (n=136, 23.7%), Hispanic (n=46, 8%), Black (n=32, 6%), and multiracial (n=31, 5%; Table 1); 64.8% of respondents identified as male and 34.3% identified as female. Most respondents were between the ages of 25 and 30 years (76.5%; Table 1).

**Table 1:** Demographics of study participants.

Characteristics		Participants, n (%)
**Gender**		
	Female	197 (34.3)
	Male	372 (64.8)
	Gender variant or nonconforming	1 (0)
	Prefer not to respond	4 (1)
**Age (years)**		
	Younger than 25	24 (4)
	25-30	439 (76.5)
	31-35	78 (14)
	36-40	23 (4)
	Older than 40	8 (1)
**Race and ethnicity**		
	Non-Hispanic White	288 (50.3)
	Asian	136 (23.7)
	Hispanic	46 (8)
	Black	32 (6)
	Multiracial	31 (5)
	Native Hawaiian or Pacific Islander	4 (1)
	Unknown	3 (1)
	Native American	2 (0)

### Influence of Social Media Presence

**Figure 1:**
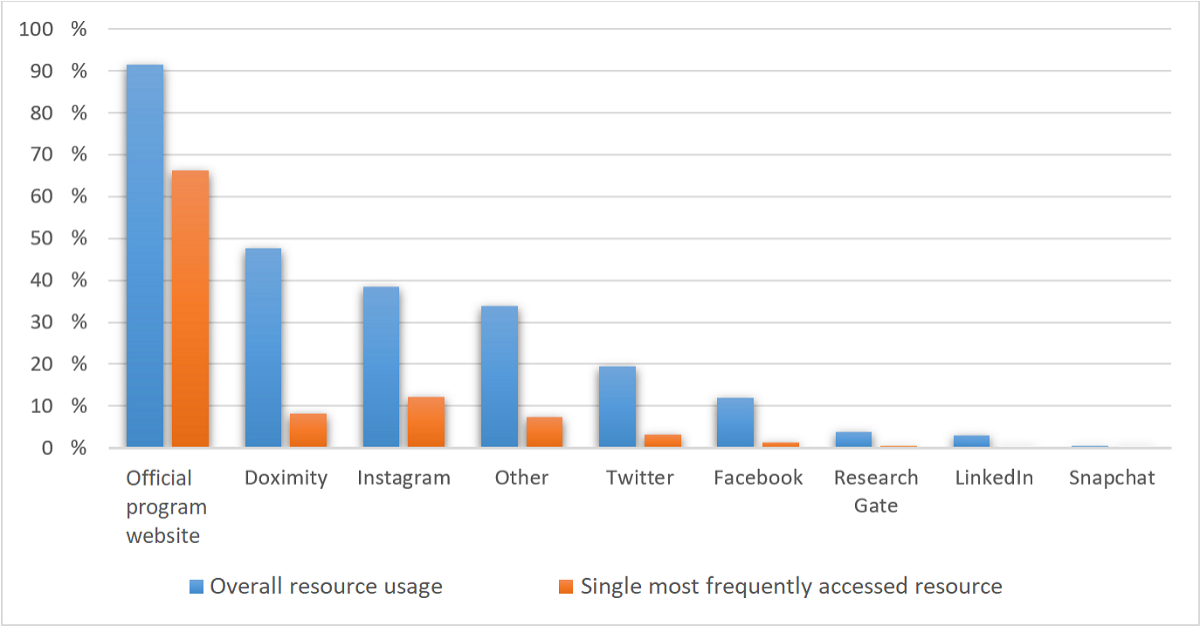
Social media and web-based resources used by applicants.

Official residency program websites were the most used resource by applicants (n=594, 91.5%), followed by Doximity (n=309, 47.6%), Instagram (n=250, 38.5%), Twitter (n=126, 19.4%), and Facebook (n=78, 12%; Figure 1).

The availability, effectiveness, and ability of social media pages to impact the applicant’s perceptions are shown in Table 2. A total of 429 (68.8%) respondents reported that social media accounts were available for at least half of the programs in which they were interested in applying to. Of the 429 respondents, 144 (23.1%) reported they were available for 75%-90% of programs they were looking at, and 53 (8%) reported they were available for greater than 90% of programs. Most respondents (64.2%) either somewhat or strongly agreed that social media pages were widely available and accessible. A majority of respondents (67.3%) reported that social media is an effective way to inform applicants about the residency program. Furthermore, 56.4% of respondents strongly or somewhat agreed that social media presence impacted their perception of the program.

Overall, applicants reported that social media positively impacted their opinion of a program, specifically 51.6% agreed that social media improved the professional image of a program and 34.6% agreed that a social media presence improved a program’s perceived prestige. Further, 73.9% (n=422) of applicants agreed that social media helps exhibit a program’s sense of culture and camaraderie and 63.8% (n=365) of them agreed that it improves a program’s transparency. Applicants reported that due to COVID-19 pandemic limitations, social media has had a significant impact on the perception of programs for 56.4% (n=320) of respondents. One-third of applicants believed that social media presence would continue to be an important factor for future application cycles, while a third believed that social media will have less of an impact when not limited by COVID-19 pandemic safety measures (Table 2).

**Table 2:** Applicants ranking of social media impact.

	Strongly agree, n (%)	Somewhat agree, n (%)	Neither agree nor disagree, n (%)	Somewhat disagree, n (%)	Strongly disagree, n (%)
Pages available and accessible	108 (18.9)	259 (45.3)	135 (23.6)	54 (9)	16 (3)
Effective way to inform applicants	152 (26.6)	233 (40.7)	117 (20.5)	53 (9)	17 (3)
Impact on perception of program	123 (21.7)	197 (34.7)	159 (28.0)	44 (8)	45 (8)
Positive impact on opinion of program	121 (21.2)	207 (36.3)	203 (35.6)	20 (3)	20 (3)
Improved programs professional image	119 (20.8)	176 (30.8)	224 (39.2)	38 (7)	14 (2)
Improved perception of programs prestige	63 (11)	135 (23.6)	274 (47.9)	62 (11)	38 (7)
Helps exhibit programs culture and camaraderie	228 (39.9)	194 (34.0)	123 (21.5)	15 (3)	11 (2)
Improved programs transparency	149 (26.0)	216 (37.8)	158 (27.6)	30 (5)	19 (3)
Due to the COVID-19 pandemic, social media will have significant impact on the perception of programs	170 (29.8)	207 (36.3)	125 (21.9)	52 (9)	17 (3)
Social media will have less of an impact on applicant during future interview cycles not limited by the COVID-19 pandemic	42 (7)	149 (26)	188 (32.9)	164 (28.7)	29 (5)

### Perceptions of Social Media by Gender, Race, and Ethnicity

Survey items were stratified by gender, race, and ethnicity, with significant differences based on gender in 20% of questions and based on race and ethnicity in 30% of questions (Multimedia Appendix 3). There were differences in the perception of social media by gender; 76.3% of female applicants strongly or somewhat agreed that social media was an effective way to inform applicants and improvement in program transparency compared to 65.2% of male applicants (*χ*^2^_1_=21.7; *P*=.041). In regards to race and ethnicity, there were differences including social media’s impact on the perception of program, positive impact of opinion of program and 80.1% of racial and ethnic minority respondents strongly or somewhat agreed social media helped exhibit programs culture and camaraderie compared to 67% of non-Hispanic White applicants (*χ*^2^=15.0; *P*=.005).

An 8-item scale with good reliability was created, representing the importance of social media (α=.838). An SEM was used to examine the relationship between race, age, and gender and the importance of social media scale; the SEM had acceptable fit indices (CFI/Tucker-Lewis Fit Index =0.97; RMSEA=0.066; SRMR=0.048). There was a positive and statistically significant relationship such that male applicants (standardized β=.151; *P*=.002) and older applicants (β=.159; *P*<.001) had less trust and reliance on social media for information regarding anesthesiology residency programs. The applicant’s race and ethnicity were not associated with the social media scale (β=–.089; *P*=.079).

### 2020-2021 Residency Application Cycle

**Figure 2:**
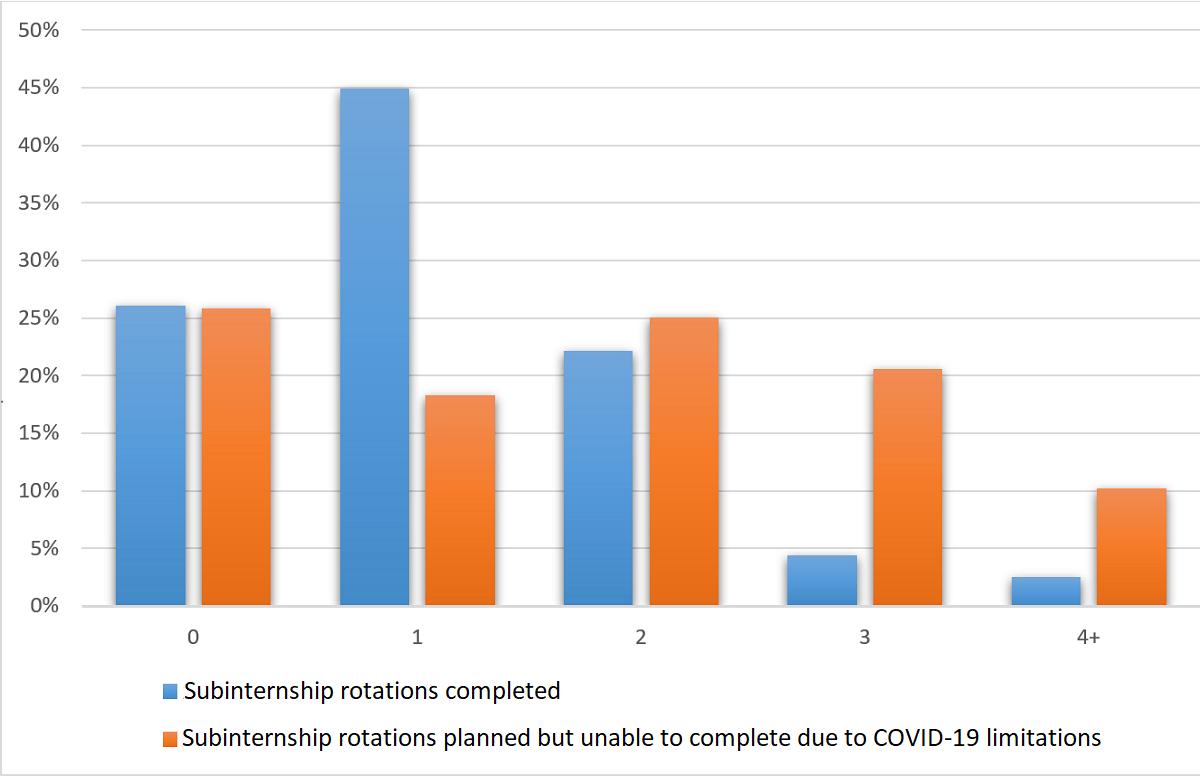
2020-2021 Subinternship rotations completed by applicants.

Figure 2 displays the results of subinternship completion versus planned completion during the 2020-2021 residency application cycle. The most common number of subinternships completed was 1 (45%), over a quarter (26.1%) of applicants did not complete a subinternship and 22.2% completed 2 subinternships. A quarter of the applicants (25.1%) planned to complete 2 subinternships but were unable to, owing to COVID-19 pandemic limitations.

## Discussion

### Principal Findings

The use of social media by residency programs has been increasing in recent years with more digital platforms to share information [6,12]. However, the impact of these platforms on applicants is not well understood. Prior studies suggest that social media can influence applicants’ perceptions and help guide their decision-making during the ranking of prospective programs [6,11-15]. Our results confirm that social media has an influence on anesthesiology residency applicants’ perception of anesthesiology residency program, and their perceptions are generally positive. According to our results, social media has been an effective way for programs to inform applicants, as well as, help display the program’s culture, camaraderie, professionalism, and transparency. Hence, anesthesiology residency programs can use social media to effectively inform and attract future applicants [16]. Residency programs should consider investing time and resources into a social media presence as it is a crucial factor for a strong recruitment effort, especially for the modern-day residency applicant [11,13,14,16,17]. This is especially salient given the circumstances of the global COVID-19 pandemic and its impact on the recruiting process but also will hold true in the future.

Social media usage by applicants and residency programs is important in determining its overall use. In our study, most applicants found social media resources for over half of their preferred anesthesiology programs. Furthermore, over a quarter of applicants reported that social media was accessible for greater than 75% of programs. These findings highlight the increased usage of social media in recent years as previous reports found that 15% of residency programs have a social media presence [9]. There are multiple factors contributing to this increase such as increased awareness of social media presence by program leadership, efforts to replicate strategies from other programs, and a compensatory mechanism from the lack of in-person interaction due to the COVID-19 pandemic. The increased use of social media by anesthesiology programs coincides with its use by applicants. In 2014, it was found that half of residency applicants use 1 or more forms of social media to learn about programs [8]. In our study, Doximity and Instagram were the most popular social media platforms, not including the official website posted by programs. It may be that applicants rely more on social media platforms to gather more information about programs that are typically not found on official program websites and programs are responding with increased social media presence. This trend overall suggests that social media’s impact and role on the residency application process will continue to grow.

This study also aimed to identify which social media platform was most valuable to applicants. Although Doximity and official residency pages were the most used resource, the use of Instagram by applicants to evaluate residency programs has increased, and this finding is consistent with a prior report [12]. Both Doximity and Instagram were reportedly used more than Twitter. Applicants rated posts that displayed social events or camaraderie among the residents in a program to be the most helpful. These results are consistent with previous observational studies that show social media can help programs display their personality and appear more approachable to applicants [12]. The combination of findings from this study and prior studies suggests that investing resources into developing an Instagram presence for programs tailored to displaying social events and resident camaraderie to be most attractive for applicants.

It is obvious that the COVID-19 pandemic has forced significant adaptations to be made in graduate medical education and residency recruitment. Programs have shifted to virtual events under the recommendation of the Accreditation Council for Graduate Medical Education [18]. The lack of in-person interviewing, coupled with the reduction of away rotations available for prospective anesthesiology residents to evaluate programs, establish connections, and make strong first impressions at programs of interest, has also contributed to a significant paradigm shift in recruitment efforts [15,19,20]. Due to these factors, applicants are likely forced to rely on virtual and digital means to not only learn more about programs but also interact with programs [15-17]. Most applicants either disagreed or felt neutral that there would be a diminished impact of social media in future cycles, suggesting that social media will continue to play a significant role in the residency application process as it relates to anesthesiology. There was a trend with male and older applicants of less trust in social media, but overall, there is consensus among the applicants that it is a beneficial tool. This finding consistent with other studies in the literature across multiple specialties. Furthermore, social media may continue to play a strong role in future cycles as it has been suggested that virtual interviews can improve residency cycle outcomes even outside of the context of COVID-19 pandemic restrictions [16,18].

Several limitations should be discussed. Our results could have been affected by response bias inherent to surveys and depend on truthful reporting by applicants (ie, social desirability bias). Despite our robust sample size, this study was limited to only those applying to a single anesthesiology residency program and does not encompass the complete anesthesiology applicant pool and thus may not be fully representative of the entire group. However, we feel that this is a strong representative sample as 55% of all medical students in the United States, who applied to anesthesiology programs, applied to this residency program and we had a response rate of 58.6%. When comparing the demographics of survey respondents and current anesthesiology residents, our study respondents strongly represent the current anesthesiology resident demographics in terms of gender, race, and ethnicity.

### Conclusions

During the 2020-2021 anesthesiology residency application cycle, many applicants were unable to complete away rotations due to COVID-19 restrictions. As a result, social media played a significant role in applicants' perception of programs. It was an effective means to inform applicants and generally positively impacted applicants' perception of programs. Aside from the traditional official website, applicants used social media platforms like Instagram to gather insight into a program’s culture and transparency, with social event posts being the most successful at engaging the applicant’s interest. The majority of applicants believed that social media would continue to be impactful in future residency application cycles not limited by COVID-19 pandemic restrictions. Thus, anesthesiology residency programs should consider investing time and resources toward building a social media presence as it is an important factor toward the recruitment of potential anesthesiology residency applicants.

## Appendix

Multimedia Appendix 1 Survey.

Multimedia Appendix 2 CHERRIES checklist.

Multimedia Appendix 3 Survey items stratified by gender, race, and ethnicity.

## Abbreviations

CFI: comparative fit index
CHERRIES: Checklist for Reporting Results of Internet e-Surveys
RMSEA: root-mean-square error of approximation
SEM: structural equation model

## Data Availability

The data sets generated and analyzed during this study are available from the corresponding author on reasonable request.
