# Supplementary material for: Influence of Social Media on Applicant Perceptions of Anesthesiology Residency Programs During the COVID-19 Pandemic: Quantitative Survey
Source: JMIR Med Educ. 2023 Jun 29;9:e39831. doi: 10.2196/39831 (PMC10337370; doi:10.2196/39831)
Supplement: Multimedia Appendix 1 [file mededu_v9i1e39831_app1.pdf]

General Questions
Q1: Which specialty are you applying to?
Q2: How many sub-internship rotations in your desired specialty will you complete this cycle?<br>• 0<br>• 1<br>• 2<br>• 3<br>• 4+
Q3: How many sub-internship rotations did you plan to complete but were not able to due to covid-19 limitations?<br>• 0<br>• 1<br>• 2<br>• 3<br>• 4+
Q4: Residency-based social media pages were available for approximately what percentage of programs in which you were interested?<br>• >90% of programs<br>• 75-90%<br>• 50-74%<br>• 25-49%<br>• Less than 25% of programs
Q5: Which of the following resources did you use to learn about residency programs? (Select all that apply)<br>• Doximity<br>• Facebook<br>• Twitter<br>• Instagram<br>• Snapchat<br>• LinkedIn<br>• Research Gate<br>• Official Residency Program Website<br>• Other
Q6: Which social media platform did you most frequently use to learn about residency programs? (Select only one)

- Doximity
- Facebook
- Twitter
- Instagram
- Snapchat
- LinkedIn
- Research Gate
- Official Residency Program Website
- Other

Agree/Disagree Questions[1]

Q7: Residency-based social media pages were widely available and easily accessible for me as an applicant.

Q8: Residency-based social media pages are an effective way to inform applicants about the associated residency program.

Q9: Residency-based social media pages had an impact on my perception of the associated program.

Q10:Residency-based social media accounts positively impacted my opinion of the program.

Q11: The presence of a residency-based social media account improved the program's professional image.

Q12: The presence of a residency-based social media account improved my perception of a program's prestige.

Q13: The presence of a residency-based social media account helped programs exhibit their culture and camaraderie among residents, faculty and staff.

Q14: The presence of a residency-based social media account made the program appear more transparent.

Q15: Social media will have a significant impact on applicant perceptions of programs during the current residency cycle due to Covid-19 limitations (i.e. Lack of in person interviews).

Q16: Social media accounts will have less of an impact on applicant perceptions during future application cycles not limited by Covid-19 safety measures (i.e. Lack of in person interviews).

Q17: Please rank the following in terms of which types of social media posts were most helpful in learning about the associated residency program: (click and drag into correct order)

- Social events/camaraderie

- Research Production
- Education (conferences, didactics, dissections, etc.)
- Faculty or resident biographical posts
- Resources or facilities
- Perks of residency geographical location
- Highlighting resident hobbies

Demographic Questions

Q18: What is your age?

- Less than 25
- 35-30 years
- 31-35 years
- 36-40 years
- Greater than 40
- Prefer not to respond

Q19: Which gender identity do you most closely identify with?

- Female
- Male
- Transgender female
- Transgender male
- Gender variant/non-conforming
- Other
- Prefer not to respond

Q20: What is your race/ethnicity?

- Black
- Native American
- Alaskan Native
- White
- Asian
- Native Hawaiian/Pacific Islander
- Ethnic origin Hispanic (any race)
- Multiracial
- Unknown
- Prefer not to respond

[1]Responses options included: strongly agree, somewhat agree, neither agree nor disagree, somewhat disagree, strongly disagree
