# Supplementary material for: Influence of Social Media on Applicant Perceptions of Anesthesiology Residency Programs During the COVID-19 Pandemic: Quantitative Survey
Source: JMIR Med Educ. 2023 Jun 29;9:e39831. doi: 10.2196/39831 (PMC10337370; doi:10.2196/39831)
Supplement: Multimedia Appendix 2 [file mededu_v9i1e39831_app2.pdf]

CHERRIES Checklist
The Influence of Social Media on Applicant Perceptions of Anesthesiology Residency Programs during the COVID-19 Pandemic

Item Category	***Checklist Item***	***Page in Manuscript***
**Design**		
	Describe survey design	6
**IRB (Institutional Review Board) approval and informed consent process**		
	IRB approval	6
	Informed consent	6
	Data protection	6
**Development and pre-testing**		
	Development and testing	6
**Recruitment process and description of the sample having access to the questionnaire**		
	Open survey versus closed survey	6
	Contact mode	6
	Advertising the survey	6
**Survey administration**		
	Web/E-mail	7
	Mandatory/voluntary	7
	Incentives	7
	Time/Date	7
	Randomization of items or questionnaires	7
	Adaptive questioning	7
	Number of Items	7
	Number of screens (pages)	7

Item Category	***Checklist Item***	***Page in Manuscript***
	Completeness check	7
	Review step	7
**Response rates**		
	Unique site visitor	7
**Preventing multiple entries from the same individual**		
	Cookies used	7
	IP check	7
	Registration	7
**Analysis**		
	Handling of incomplete questionnaires	7
	Questionnaires submitted with an atypical timestamp	7
	Statistical correction	7
