# Supplementary material for: Influence of Social Media on Applicant Perceptions of Anesthesiology Residency Programs During the COVID-19 Pandemic: Quantitative Survey
Source: JMIR Med Educ. 2023 Jun 29;9:e39831. doi: 10.2196/39831 (PMC10337370; doi:10.2196/39831)
Supplement: Multimedia Appendix 3 [file mededu_v9i1e39831_app3.pdf]

Appendix 3: Survey items stratified by Gender and Race/Ethnicity

Survey Question	Response	Gender Total, n (%)	Female, n (%)	Male, n (%)	Race Total, n (%)	White, n (%)	Non-White, n (%)
**Pages Available and Accessible**	Strongly Agree	90 (18.9)	31 (18.8)	59 (19.2)	99 (18.6)	47 (17.5)	52 (19.7)
**Gender χ2:** 7.64, **p:** 0.812	Somewhat Agree	221 (46.5)	84 (50.9)	135 (44)	239 (44.9)	124 (46.3)	115 (43.6)
**Race χ2:** 2.06, **p:** 0.724	Neither Agree or Disagree	101 (21.3)	26 (15.8)	74 (24.1)	126 (23.7)	66 (24.6)	60 (22.7)
	Somewhat Disagree	51 (10.7)	20 (12.1)	31 (10.1)	54 (10.2)	26 (9.7)	28 (10.6)
	Strongly Disagree	12 (2.5)	4 (2.4)	8 (2.6)	14 (2.6)	5 (1.9)	9 (3.4)
**Effective Way to Inform Applicants**	Strongly Agree	127 (26.7)	57 (34.5)	69 (22.5)	138 (25.9)	57 (21.3)	81 (30.6)
**Gender χ2: 21.69, p: 0.041**	Somewhat Agree	200 (42.1)	69 (41.8)	131 (42.7)	215 (40.3)	115 (42.9)	100 (37.7)
**Race χ2:** 6.32, **p:** 0.176	Neither Agree or Disagree	93 (19.6)	24 (14.5)	68 (22.1)	114 (21.4)	61 (22.8)	53 (20)
	Somewhat Disagree	43 (9.1)	14 (8.5)	28 (9.1)	51 (9.6)	28 (10.4)	23 (8.7)
	Strongly Disagree	12 (2.5)	1 (0.6)	11 (3.6)	15 (2.8)	7 (2.6)	8 (3)
**Impact on Perception of Program**	Strongly Agree	107 (22.5)	46 (27.9)	60 (19.5)	115 (21.7)	46 (17.2)	69 (26.1)
**Gender χ2:** 16.58, **p:** 0.166	Somewhat Agree	168 (35.4)	58 (35.2)	110 (35.8)	180 (33.9)	87 (32.6)	93 (35.2)
**Race χ2: 13.55, p: 0.009**	Neither Agree or Disagree	128 (26.9)	39 (23.6)	88 (28.7)	151 (28.4)	78 (29.2)	73 (27.7)
	Somewhat Disagree	37 (7.8)	14 (8.5)	22 (7.2)	42 (7.9)	28 (10.5)	14 (5.3)
	Strongly Disagree	35 (7.4)	8 (4.8)	27 (8.8)	43 (8.1)	28 (10.5)	15 (5.7)

**Positive Impact on Opinion of Program**	Strongly Agree	102 (21.5)	44 (26.7)	57 (18.6)	111 (20.9)	49 (18.3)	62 (23.5)
**Gender χ2:** 19.1, **p:** 0.087	Somewhat Agree	177 (37.3)	72 (43.6)	104 (33.9)	190 (35.7)	85 (31.7)	105 (39.8)
**Race χ2: 10.44, p: 0.034**	Neither Agree or Disagree	164 (34.5)	40 (24.2)	123 (40.1)	104 (36.5)	111 (41.4)	83 (31.4)
	Somewhat Disagree	16 (3.4)	5 (3)	11 (3.6)	19 (3.6)	13 (4.9)	6 (2.3)
	Strongly Disagree	16 (3.4)	4 (2.4)	12 (3.9)	18 (3.4)	10 (3.7)	8 (3)
**Improved Programs Professional Image**	Strongly Agree	98 (20.6)	34 (20.6)	63 (20.5)	109 (20.5)	49 (18.3)	60 (22.6)
**Gender χ2:** 12, **p:** 0.446	Somewhat Agree	147 (30.9)	56 (33.9)	91 (29.6)	162 (30.4)	72 (26.9)	90 (34)
**Race χ2:** 8.65, **p:** 0.07	Neither Agree or Disagree	188 (39.6)	64 (38.8)	123 (40.1)	212 (39.8)	123 (45.9)	89 (33.6)
	Somewhat Disagree	34 (7.2)	9 (5.5)	24 (7.8)	38 (7.1)	18 (6.7)	20 (7.5)
	Strongly Disagree	8 (1.7)	2 (1.2)	6 (2)	12 (2.3)	6 (2.2)	6 (2.3)
**Improved Perception of Programs Prestige**	Strongly Agree	48 (10.1)	15 (9.1)	32 (10.4)	54 (10.1)	24 (8.9)	30 (11.4)
**Gender χ2:** 13.2, **p:** 0.353	Somewhat Agree	115 (24.2)	41 (24.8)	74 (24.1)	125 (23.4)	55 (20.4)	70 (26.5)
**Race χ2:** 6.52, **p:** 0.163	Neither Agree or Disagree	228 (48)	84 (50.9)	142 (46.3)	263 (49.3)	147 (54.4)	116 (43.9)
	Somewhat Disagree	55 (11.6)	15 (9.1)	40 (13)	57 (10.7)	26 (9.6)	31 (11.7)
	Strongly Disagree	29 (6.1)	10 (6.1)	19 (6.2)	35 (6.6)	18 (6.7)	17 (6.4)
**Helps Exhibit Programs Culture and Camaraderie**	Strongly Agree	201 (42.3)	85 (51.5)	115 (37.5)	212 (39.8)	104 (38.5)	108 (41.2)

**Gender χ2:** 16.28, **p:** 0.179	Somewhat Agree	162 (34.1)	55 (33.3)	106 (34.5)	179 (33.6)	77 (28.5)	102 (38.9)
**Race χ2: 15.04, p: 0.005**	Neither Agree or Disagree	91 (19.2)	21 (12.7)	69 (22.5)	117 (22)	74 (27.4)	43 (16.4)
	Somewhat Disagree	14 (2.9)	3 (1.8)	11 (3.6)	15 (2.8)	11 (4.1)	4 (1.5)
	Strongly Disagree	7 (1.5)	1 (0.6)	6 (2)	9 (1.7)	4 (1.5)	5 (1.9)
**Improved Programs Transparency**	Strongly Agree	133 (28)	56 (33.9)	77 (25.1)	140 (26.3)	67 (24.9)	73 (27.7)
**Gender χ2: 22.86, p: 0.029**	Somewhat Agree	178 (37.5)	68 (41.2)	109 (35.5)	197 (37)	92 (34.2)	105 (39.8)
**Race χ2:** 6.97, **p:** 0.138	Neither Agree or Disagree	122 (25.7)	27 (16.4)	94 (30.6)	150 (28.1)	88 (32.7)	62 (23.5)
	Somewhat Disagree	28 (5.9)	10 (6.1)	17 (5.5)	29 (5.4)	12 (4.5)	17 (6.4)
	Strongly Disagree	14 (2.9)	4 (2.4)	10 (3.3)	17 (3.2)	10 (3.7)	7 (2.7)
**Due to COVID-19, social media will have significant impact on perception of programs.**	Strongly Agree	155 (32.6)	69 (41.8)	85 (27.7)	163 (30.6)	67 (25)	96 (36.4)
**Gender χ2:** 16.58, **p:** 0.166	Somewhat Agree	167 (35.2)	56 (33.9)	110 (35.8)	185 (34.8)	97 (36.2)	88 (33.3)
**Race χ2:** 9.02, **p:** 0.061	Neither Agree or Disagree	100 (21.1)	24 (14.5)	75 (24.4)	120 (22.6)	66 (24.6)	54 (20.5)
	Somewhat Disagree	43 (9.1)	12 (7.3)	31 (10.1)	49 (9.2)	29 (10.8)	20 (7.6)
	Strongly Disagree	10 (2.1)	4 (2.4)	6 (2)	15 (2.8)	9 (3.4)	6 (2.3)
**Social media will have less of an impact on applicant during**	Strongly Agree	35 (7.4)	8 (4.8)	27 (8.8)	38 (7.1)	20 (7.4)	18 (6.8)

**future interview cycles not limited by COVID-19.**							
**Gender χ2:** 13.73, **p:** 0.318	Somewhat Agree	119 (25.1)	32 (19.4)	87 (28.3)	131 (24.6)	71 (26.3)	60 (22.8)
**Race χ2:** 8.30, **p:** 0.081	Neither Agree or Disagree	151 (31.8)	53 (32.1)	97 (31.6)	180 (33.8)	94 (34.8)	86 (32.7)
	Somewhat Disagree	147 (30.9)	62 (37.6)	83 (27)	156 (29.3)	78 (28.9)	78 (29.7)
	Strongly Disagree	23 (4.8)	10 (6.1)	13 (4.2)	28 (5.3)	7 (2.6)	21 (8)
